# PS/PANI/MoS_2_ Hybrid Polymer Composites with High Dielectric Behavior and Electrical Conductivity for EMI Shielding Effectiveness

**DOI:** 10.3390/ma12172690

**Published:** 2019-08-22

**Authors:** Abdul Saboor, Saad Mahmood Khalid, Rahim Jan, Ahmad Nawaz Khan, Tanveer Zia, Muhammad Umer Farooq, Shaista Afridi, Muhammad Sadiq, Muhammad Arif

**Affiliations:** 1 School of Chemical and Materials Engineering, National University of Sciences and Technology, Sector H-12, Islamabad 46000, Pakistan; 2 U.S-Pakistan Center for Advance Studies in Energy, University of Engineering & Technology, Peshawar 25000, Khyber PakhtunKhwa, Pakistan; 3 Mechanical Engineering Department, University of Engineering & Technology, Peshawar 25000, Khyber Pakhtunkhwa, Pakistan

**Keywords:** polymer blends, MoS_2_ nanosheets, dielectric characteristics, EMI Shielding

## Abstract

Liquid exfoliated molybdenum disulfide (MoS_2_) nanosheets and polyaniline (PANI) nanoparticles are dispersed in polystyrene (PS) matrix to fabricate hybrid polymer composites with high dielectric and electromagnetic interference (EMI) shielding behavior. A phase-separated morphology is formed when PANI and MoS_2_ are incorporated into polystyrene (PS) matrix. An increasing concentration of MoS_2_ nanoparticles inside PS/PANI (5 wt %) polymer blend forms an interconnected network, resulting in high electrical conductivity and dielectric behavior, making them a suitable candidate for EMI shielding application. An increment in dielectric constant and loss, up to four and five orders of magnitude, respectively, is recorded at a maximum concentration of 1 wt % of MoS_2_ in PS/PANI-5 polymer blend at 100 Hz. The enhanced dielectric characteristics for PS/PANI/MoS_2_ composites are then theoretically evaluated for the estimation of EMI shielding effectiveness in the frequency range of 100 Hz to 5 MHz. The maximum dielectric constant and loss achieved for PS/PANI-5 wt %/MoS_2_-1 wt % are responsible for estimated shielding effectiveness of around 92 dB at 100 Hz. The increase in dielectric behavior and shielding effectiveness is probably due to the increased number of charged dipoles accumulated at the insulator–conductor interface.

## 1. Introduction

Electromagnetic interference (EMI) has become a major problem with the exponential growth of more small and functional electronic devices. Electronic components (such as high speed processors) produce unwanted electromagnetic energy, affecting the performance and lifetime of other such components by interference [1,2,3]. It may cause loss of stored data, and temporary as well as permanent disruption of important functions. Moreover, a long-time exposure of living things to an environment filled with excessive EM pollution increases the risk of diseases [4,5]. In order to reduce this pollution, researchers are working extensively to produce materials having both conductive as well as magnetic behavior, which could shield EM waves [6,7,8,9,10]. Primarily, metals and magnets were used to reduce the EM pollution. But due to a number of problems, such as difficult processing, handling, and high cost, a new set of electrically conductive polymer composites were focused. These polymer composites containing various nano fillers such as ferroelectric ceramics, layered silicates, carbon nanotubes, graphene, etc., are very promising [11,12,13,14,15,16,17,18,19,20]. Carbon-based polymer composites are cost effective and easy to process, previously studied by a number of researchers [21,22,23,24,25,26], but to make them more suitable for EM shielding, other avenues are explored to make full use of synergetic properties by adding new fillers [27]. 

In recent years, polyaniline (PANI), a highly conductive semi-crystalline polymer has been studied extensively for EMI shielding applications, but due to its poor mechanical properties, PANI cannot be used as a single system. Therefore, PANI is used as filler in a suitable polymer matrix to prepare highly conducive polymer blends, which in turn can be used for EMI shielding and many more applications. We recently [28] prepared highly conductive SAN/PANI polymer blends via a solution casting technique with varying concentrations of PANI in styrene acrylonitrile (SAN) polymer matrix. The increase in alternating current (AC) conductivity and dielectric constant was around ten and five orders of magnitude at 1 kHz by the addition of 40 wt % of PANI in styrene acrylonitrile, respectively. The increase in dielectric and AC conductivity translated in to increased total shielding efficiency of 164 dB at 1 kHz at 40 wt % concentration of PANI. Similarly, M.C. Arenas et al. [29] described how polymer nanocomposites PVA/PANI, doped with SDS have increased AC conductivity up to 0.044 S/cm at low loading of 5 wt % PANI in PVA matrix. To make polymer composites more conductive or dielectric in nature, a third component of nanofillers may also be added. The experimental discovery of graphene has revived the two-dimensional (2D) materials family with more exotic materials to follow. The exponential growth in the field of nanoelectronics and optoelectronics has opened up new research interest, which is to replace graphene with a new set of 2D materials. Molybdenum disulfide MoS_2_ is one such 2D material having a layered structure similar to that of graphene. Electrons in MoS_2_ are normal fermions with parabolic energy dispersion, and are semiconductors with a finite band gap of 1.2 eV, prior to any gap-opening engineering, which makes MoS_2_ a more competitive candidate than graphene to be used in devices with increase dielectric constant and AC conductivity consequently improving EMI shielding application [30]. The individual crystalline layers of MoS_2_ are stacked above each other, having a thickness of 0.65 nm, held together by weak van der wall forces [31,32]. MoS_2_ has been used as a nanofiller in combination with different polymers and other nanomaterials for various applications, including EMI shielding. Wang et al. [33] reported an increase in reflection loss as a function of concentration of RGO/MoS_2_ in wax matrix and thickness of the sample, respectively. At low loading of only 10 wt % of RGO/MoS_2_ in wax matrix, a maximum reflection loss of −50.9 dB is observed at a frequency of 11.68 GHz with thickness of 2.3 mm. The introduction of PANI to polymer matrix increases AC conductivity, dielectric constant, and dielectric loss of produced polymer blend, but to produce more synergetic properties, MoS_2_ can be used as third component for maximum EMI shielding application. 

In this paper, we study how MoS_2_ can alter the nature of a polymer composite by replacing carbon-based nanofillers for EMI shielding applications. A three-phase hybrid polymer composite of polystyrene, PANI, and MoS_2_ were prepared using a solution casting technique. At optimum concentration of MoS_2_, both conductive and EMI shielding responses increase, which may be due to the formation of a conductive network of nanofillers inside polymer matrix. The average thickness of (free standing) thin films of PS/PANI/MoS_2_ hybrid polymer composites is 0.1 mm, which is considerably low. The dielectric characteristics measurements in the frequency range 100 Hz to 5 MHz are utilized for theoretical EMI shielding effectiveness evaluation. The current research will add to the hybrid polymer composite utilization for EMI shielding, especially in terms of the inclusion of MoS_2_ nanosheets, which have not been used frequently [33,34,35,36,37].

## 2. Materials and Methods

### 2.1. Materials

Polystyrene (PS) commercial grade was purchased from ERKOL, Istanbul, Turkey, whereas aniline monomers were provided by Uni CHEM chemical reagents (Beograd, Serbia), which were purified through distillation. Sodium dodecyl sulphate (SDS) was purchased from Sigma Aldrich (Czechia, Germany). Daejung chemicals, Shiheung, Korea provided ammonium peroxydisulfate (APS) (99% pure). MoS_2_ nanosheets with 3–5 layers on average were produced through liquid exfoliation method [38]. MoS_2_ contained a hydroxyl group, which assisted in uniform dispersion of MoS_2_ in polystyrene matrix. The concentration of PANI was fixed (5 wt %), while MoS_2_ concentration was varied (0.1 wt % and 1.0 wt %) in PS/PANI/MoS_2_ hybrid polymer composites. 

### 2.2. Synthesis of Polyaniline (PANI)

Conductive Polyaniline (PANI) was made via a chemical oxidation polymerization method. In the beginning, aniline monomer (ANI) (6 gm) was added in 100 mL of deionized water in a flask containing a magnetic stirrer. The whole setup was situated in an ice bath to keep the temperature at 0 °C. To make it conductive, 1 M HCL acid solution was added to the solution, which produced aniline hydrochloride after continuous stirring. Then, 1 M SDS surfactant was also added to the solution. To initiate the polymerization, an initiator APS with a concentration of 1.2 gm was dissolved in 20 gm deionized water. Slowly, the solution was added to the aniline solution at a fixed temperature of 0 °C with 1 h stirring, followed by stirring for 1.5 h at room temperature. To precipitate the polymer, methanol was used, and then distilled water was used to wash it several times, followed by drying in a vacuum oven at 50 °C. The final product was a green powder of conductive PANI.

### 2.3. Thin Film Synthesis

#### 2.3.1. PS/PANI Polymer Blend

Polymer blend thin films were prepared using a solution casting technique. PS took 40 min to dissolve in toluene through continuous stirring. Afterwards, to remove any moisture, polyaniline (PANI) powder was first dried at 60 °C in a vacuum oven, then a weighed amount of 5 wt % PANI was added to the PS solution, which was first sonicated for 1 h by probe sonicator for better mixing and then stirred for 24 h. In order to obtain the thin films of polymer blend, petri dishes were used, to which the solution was added and dried overnight in a fume hood at a room temperature. Further drying of PS/PANI polymer blends was done in a vacuum oven at 60 °C for 5 h.

#### 2.3.2. PS/PANI/MoS_2_ Hybrid Polymer Composites

To prepare PS/PANI-5/MoS_2_ hybrid polymer composites, MoS_2_ nanoparticles were added to the PS/PANI polymer blend with PANI at 5 wt % concentration. A solution casting method was used in which the required amounts of MoS_2_ nanoparticles were dispersed by stirring and sonication in the PS/PANI solution. Firstly, 1 h probe sonication was done, after which stirring of the solution was done for 24 h. For overnight drying in a fume hood, the solution was added to petri dishes at room temperature. To further remove the moisture, the samples were placed in a vacuum oven. Prepared casted thin films of hybrid polymer composites had a thickness of about 0.1 mm. The concentration of MoS_2_ nanoparticles in PS/PANI-5 wt % polymer blends varied from 0.1 to 1.0 wt %.

### 2.4. Characterization Techniques

The dimensional analysis of MoS_2_ nanosheets was carried out by AFM (Jeol SPM-5200, Tokyo, Japan), used in tapping mode. Moreover, to further study the morphology of hybrid polymer composites Analytical Scanning Electron Microscope (JOEL JSM-6490A, Tokyo, Japan) was used. To study the cross section of polymer samples, the casted films were cryogenically cracked using liquid nitrogen. To make the sample conductive, gold coating was done using an ion sputtering device (JOEL JFC-1500, Tokyo, Japan). A Theta-Theta instrument by STOE-Germany (Munich, Germany) with Cu Kα Radiations (λ = 0.15418 nm) was used to study the XRD patterns at a scanning rate of 2 °/min over the range 2Ɵ = 5–30°. The dielectric response was determined using a Wayne Kerr 6500B precision impedance analyzer (West Sussex, UK) at room temperature. The circular shaped samples of 13 mm in diameter were cut from casted films to measure their dissipation factor D and capacitance C as a function of frequency ranging from 100 Hz to 5 MHz.

## 3. Results and Discussion

### 3.1. Morphology and Structure

#### 3.1.1. Atomic Force Microscopy and Scanning Electron Microscope

**Figure 1:**
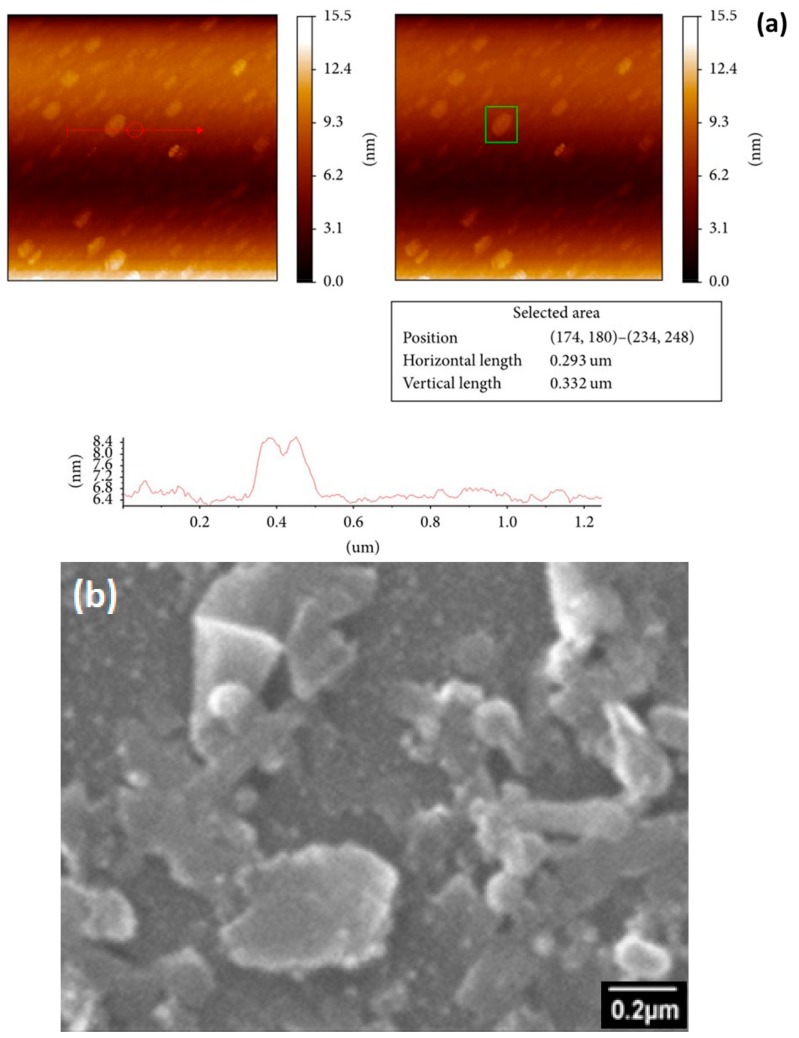
(**a**) Representative atomic force microscopy (AFM) image and dimensional analysis for molybdenum disulfide (MoS_2_) nanosheets length (L) and thickness (t). (**b**) Scanning electron micrograph of exfoliated MoS_2_ for lengths approximation.

The liquid phase exfoliation is a method that provides a relatively high yield of 2D nanosheets required for composites preparation. In this method, centrifugation is utilized for size selection of exfoliated 2D nanosheets. The basis of size selection is to change the centrifugation speed from high to low and obtain the low to high aspect ratio nanosheets [39,40,41,42]. After size selection, the dimensions of nanosheets (length: L, width: W, and thickness: t) can be measured by utilizing Raman spectroscopy, transmission electron microscopy (TEM), and atomic force microscopy (AFM) [43]. We have determined the size of MoS_2_ nanosheets in our previous work [38] by utilizing AFM (Jeol SPM 5200) in tapping mode [44], predicting L ~ 0.5–1 µm and t ~ 2 nm (3–5 layers), shown in Figure 1a. Scanning electron micrograph supported the lateral dimension claim (Figure 1b). Both these values of dimensions are highly approximated.

#### 3.1.2. Scanning Electron Microscopy of Thin Films

**Figure 2:**
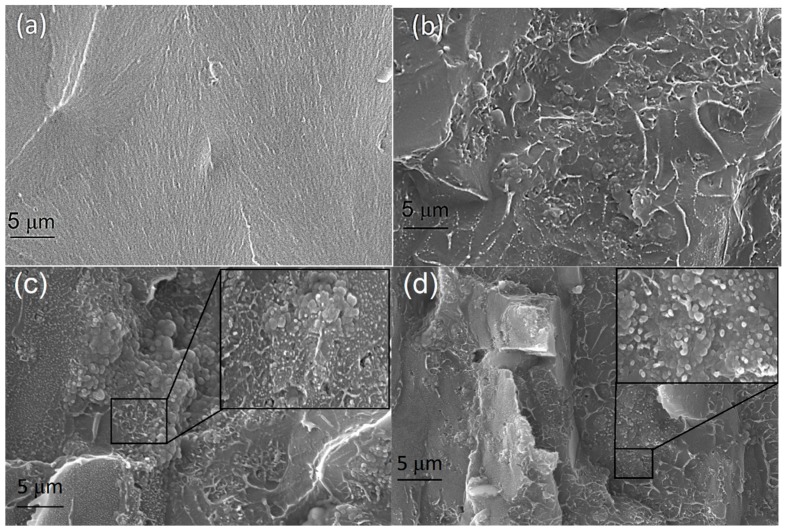
SEM micrographs of (**a**) polystyrene (PS), (**b**) PS/polyaniline (PANI)-5 wt %, (**c**) PS/PANI-5 wt %/MoS_2_-0.1 wt %, (**d**) PS/PANI-5 wt %/MoS_2_-1 wt %. The selected squared region is further zoomed in to show the dispersion of MoS_2_ at 1 μm.

Figure 2 shows the morphology of neat polystyrene, PS/PANI-5 wt % polymer blend, and PS/PANI-5/MoS_2_ (0.1 wt % and 1 wt %) hybrid polymer composites by using a scanning electron microscope. The amorphous nature of pure PS is clearly shown in Figure 2a. For PS/PANI-5 wt % polymer blends (Figure 2b), the bright lines indicate the dispersion of PANI phase in PS matrix. Images show the heterogeneous morphology of PS/PANI polymer blends, representing the immiscibility of PANI phase in PS matrix. The SEM images of hybrid polymer composites PS/PANI-5/MoS_2_-0.1 and 1 are shown in Figure 2c,d. A proper dispersion of PANI phase in PS matrix can be seen by bright lines interconnected with each other. The layer-like structure of MoS_2_ is also evident in the images displaying a uniform and random distribution of MoS_2_ in PS/PANI polymer blend. It is noteworthy that MoS_2_ layers are not only interconnected with each other but also with PANI, forming a conductive network of PANI/ MoS_2_ inside the PS polymer.

#### 3.1.3. X-ray Diffractometer

**Figure 3:**
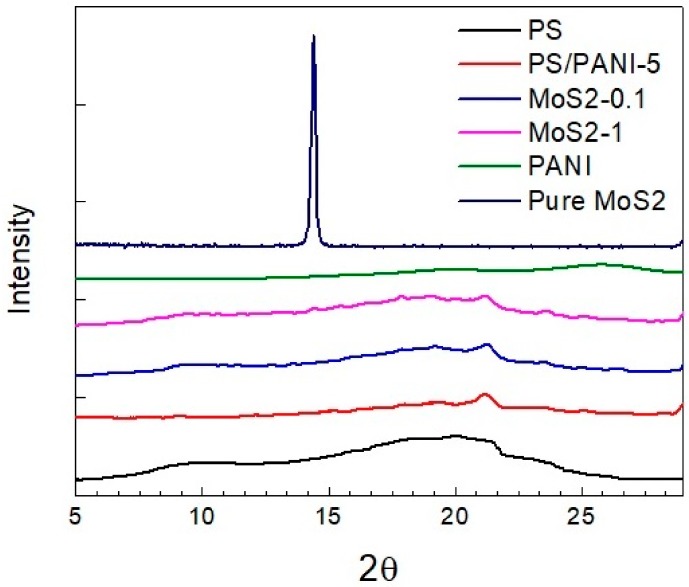
X-ray diffraction of PS, PANI, PS/PANI-5 wt % polymer blend and PS/PANI/MoS_2_ hybrid polymer composites with varying concentration of MoS_2_.

The pattern of X-ray diffraction of neat PS, neat PANI, neat MoS_2_ nanosheets, PS/PANI-5 polymer blend, and PS/PANI/MoS_2_ hybrid polymer composites with varying concentrations of MoS_2_ is shown in Figure 3. The amorphous hump of PS around 2θ = 21.3° clearly indicates its non-crystalline structure, whereas the diffraction peaks for neat PANI appears at 2θ = 15.7°, 19.7°, and 25.5°, corresponding to (011), (020), and (200) crystal planes, respectively [45]. The main X-ray diffraction peak for MoS_2_ nanosheets can be seen at 2θ = 14.4° (002), representing well-stacked layered crystalline structure. In the case of PS/PANI-5 polymer blend, the diffraction pattern shows that the main peaks of PANI disappear, representing phase-separated morphology, whereas in the hybrid polymer composite with varying concentrations of MoS_2_ (0.1–1 wt %) in PS/PANI polymer blend, the main peak has completely vanished, corresponding to a proper dispersion of MoS_2_ nanosheets in PS/PANI-5 polymer blend.

### 3.2. Dielectric Properties

**Figure 4:**
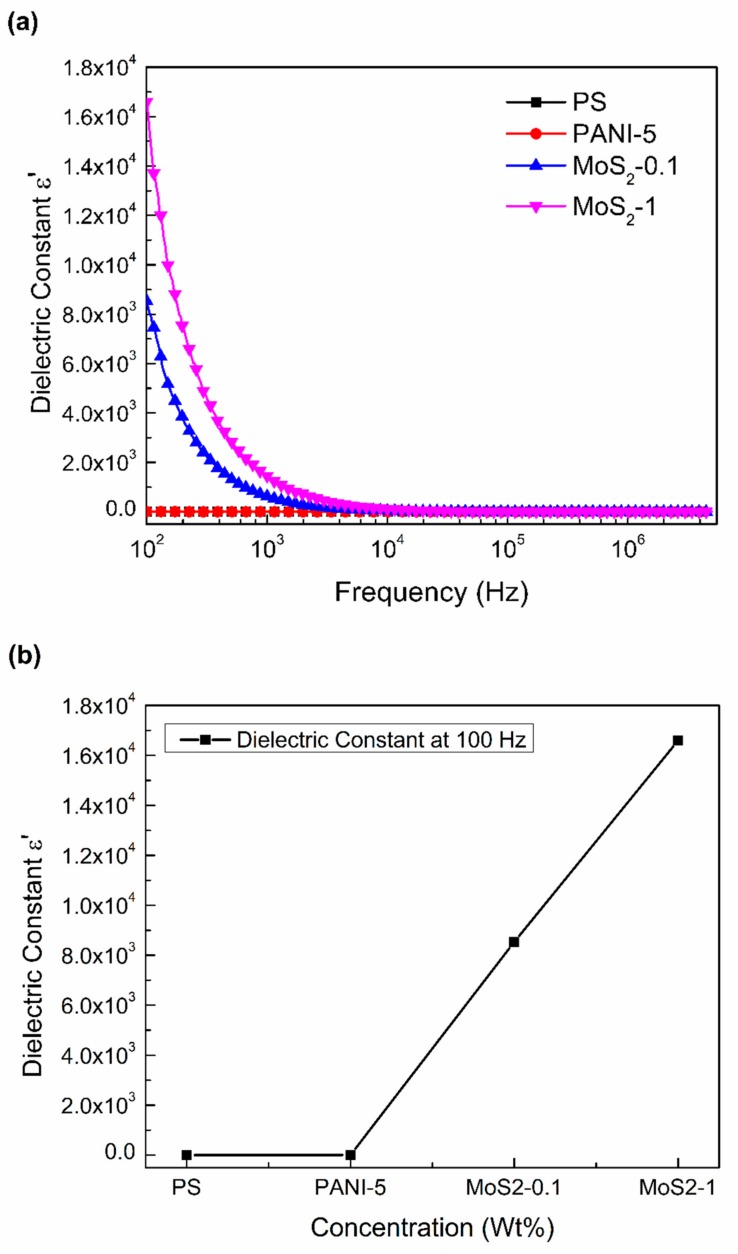
Dielectric constant as a function of frequency of (**a**) PS, PANI, PS/PANI-5 wt % polymer blend, and PS/PANI/MoS_2_ hybrid polymer composites. Dielectric constant for (**b**) PS, PANI, PS/PANI-5 wt % polymer blend, and PS/PANI/MoS_2_ hybrid polymer composites at 100 Hz.

**Figure 5:**
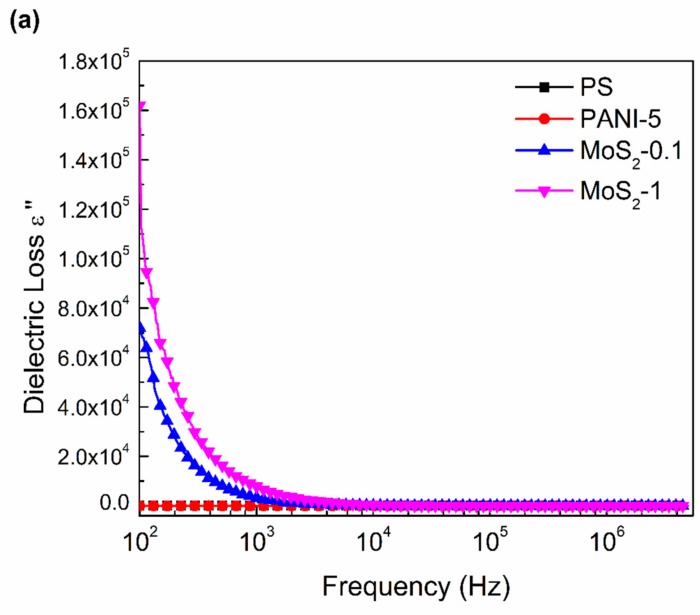

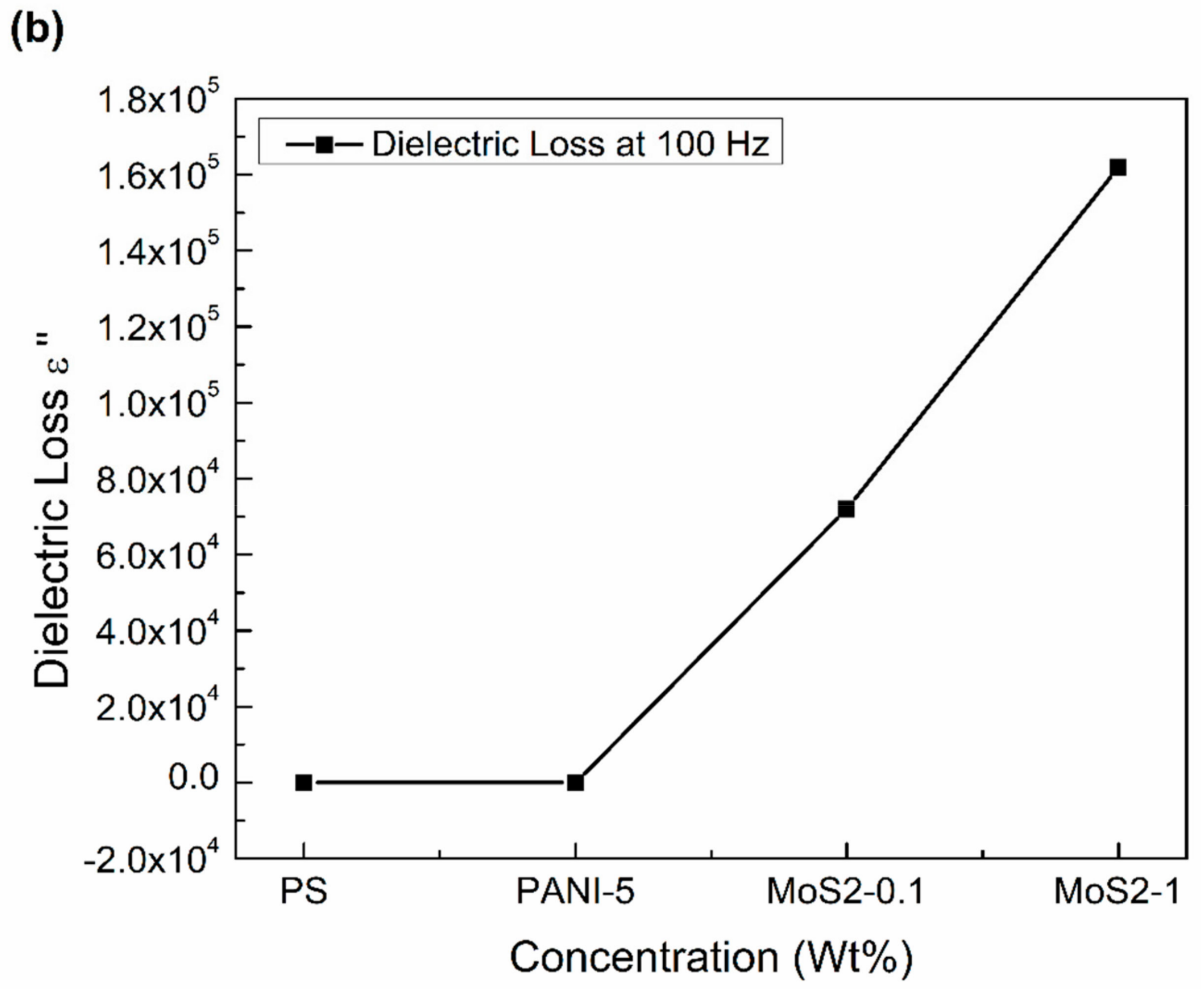
Dielectric loss versus frequency of (**a**) PS, PANI, PS/PANI-5 wt % polymer blend, and PS/PANI/MoS_2_ hybrid polymer composites. Dielectric loss for (**b**) PS, PANI, PS/PANI-5 wt% polymer blend, and PS/PANI/MoS_2_ hybrid polymer composites at 100 Hz.

**Figure 6:**
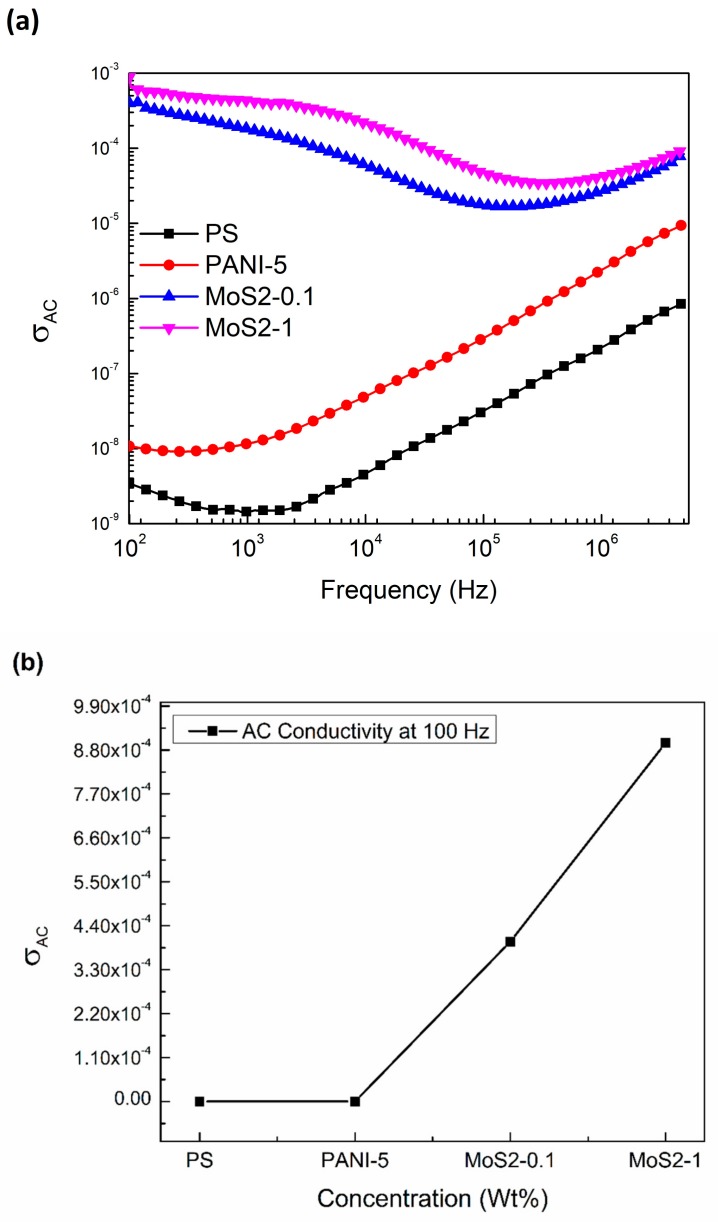
Alternating Current (AC) conductivity of (**a**) PS, PANI, PS/PANI-5 wt % polymer blend, and PS/PANI/MoS_2_ hybrid polymer composites as a function of frequency. Values of AC conductivity for (**b**) PS, PANI, PS/PANI-5 wt % polymer blend, and PS/PANI/MoS_2_ hybrid polymer composites at 100 Hz.

Figure 4, Figure 5 and Figure 6 represent the behavior of dielectric constant (*ε*′), dielectric loss (*ε*″), and AC conductivity (σ_AC_) of pure PS, PS/PANI-5 wt % polymer blend and hybrid polymer composites, respectively. The dissipation D and capacitance C were measured as a function of frequency (100 Hz to 5 MHz). Dielectric behavior is represented in terms of real (*ε*′) and imaginary (*ε*″) parts of dielectric permittivity, calculated from complex permittivity [46] represented as

*ε* = *ε*′ − *iε*” (1)

The dielectric constant (*ε*′) shows the ability of a material to store electrical energy in the electric field, which is expressed by the following mathematical expression:

(2) $$\varepsilon \prime =\frac{CD}{A\varepsilon_{o}}$$

where C is the capacitance, d is the thickness and A is the area of the sample, and *ε_o_* is free space permittivity.

The dielectric constant response is shown in Figure 4a,b as a function of alternating frequency for PS/PANI-5/MoS_2_-0.1 and 1 at room temperature. Since pure PS is an insulating polymer having no electric dipoles, it consequently shows low values of dielectric constant, i.e., almost independent of alternating frequency. The addition of PANI at 5 wt % concentration in PS slightly enhances the dielectric response at lower frequency, while at higher frequency range, the dielectric response reduces. To further enhance the dielectric constant, MoS_2_ nanosheets at 0.1 wt % and 1 wt % concentrations, respectively, were added to the PS/PANI-5 polymer blend. A rapid increase in the dielectric constant (9 × 10^3^) is observed at 0.1 wt % MoS_2_, whereas the maximum increase from 1.83 to 1.6 × 10^4^ is observed by the addition of 1 wt % MoS_2_ in PS/PANI-5 polymer blend at 100 Hz. Similar results were recently shown by Zhang et al. [47] when the dielectric constant of BiTiO_3_@carbon/silicon carbide/poly(vinylidene fluoride–hexafluoropropylene reached the maximum value of 1394 from 80 with the addition of SiC nanoparticles at 7.8 wt % concentration to BT@C-2(50 wt %)/PVDF-HFP at 1 kHz frequency. The increase in dielectric constant is almost 17 times that for the pure PVDF-HFP. The notable increase in the (*ε*′) at lower frequencies can be attributed to the Maxwell–Wagner polarization effect caused by insulator–conductor interfaces. At lower frequencies, the assembly of dipoles or charges at the conductor–insulator interface gets enough time to orient themselves according to the changing frequency, resulting in interfacial polarization enhancement. However, at higher frequency, the frequency changes so rapidly that the dipoles or space charges are unable to align themselves accordingly and therefore, the polarization is not seen.

The imaginary part of complex permittivity represents the quantification of dielectric materials inherent dissipation of electromagnetic energy. The expression to represent the dielectric loss mathematically is as followed:

*ε*″ = *ε*′tan*δ* (3)

where (*ε*″) is the dielectric loss, (*ε*′) dielectric constant, and tanδ is the dissipation factor.

Figure 5a,b shows the dielectric loss response of pure PS, PS/PANI-5 polymer blend, and PS/PANI-5/MoS_2_ hybrid polymer composites as a function of alternating frequency at room temperature. Pure PS and PS/PANI-5 Polymer response is independent of the frequency with no significant change in dielectric loss. A notable increase to 7.2 × 10^4^ occurs (100 Hz) when a 0.1 wt % MoS_2_ is added to PS/PANI-5 polymer blend. A maximum increase in of 1.6 × 10^5^ is observed at 1 wt % MoS_2_ in PS/PANI-5 as compared to 0.02 for PS only at 100 Hz. Gamal et al. [48] also studied the dielectric response of PVA/PANI polymer blend in the lower frequency range, and noted a maximum increase in both *ε*′ and *ε*″at 70 wt % PANI in PVA matrix. 

The AC conductivity of pure PS, PS/PANI-5 polymer blend, and PS/PANI-5/MoS_2_ hybrid polymer composites can be calculated by the following expression:

σ_AC_ = ω*ε_o_ ε′* tan*δ* (4)

where σ_AC_ is the AC conductivity, ω is the frequency of the applied signal, *ε_o_* is the free space permittivity, and *ε*′ is dielectric constant.

Figure 6a,b shows the AC conductivity of mentioned samples as a function of alternating frequency at room temperature. AC conductivity is increased both as a function of frequency as well as with the inclusion of MoS_2_ nanosheets in PS/PANI-5 polymer blend. At 100 Hz, both pure PS polymer and PS/PANI-5 polymer blend show a complete insulative behavior with no free charges or dipoles, which may respond to the alternating electric field. Meanwhile, an enhancement in σ_AC_ is observed at lower frequencies with the inclusion of MoS_2_ nanosheets in PS/PANI-5 polymer blend. The increase in σ_AC_ near 100 Hz indicates the contribution of interfacial polarization to induce conductivity into PS/PANI-5 polymer blend as the concentration of MoS_2_ increases. Moreover, the σ_AC_ conductivity is also enhanced with the frequency. This increase in σ_AC_ can be credited to a coping mechanism, i.e., as the strength of frequency increases, the dipoles are stretched in a way that they overlap each other, causing the charges to jump and enhancing the conductivity within the system. 

For comparison, all the values of (*ε*′), (*ε*″), and σ_AC_ for PS, PS/PANI-5, and PS/PANI-5/MoS_2_ hybrid polymer composites at 100 Hz are listed in Table 1.

Overall, a notable enhancement is observed in the dielectric properties with the addition of MoS_2_ nanosheets in PS/PANI-5 polymer blend. The addition of PANI at 5 wt % concentration in pure PS polymer does not have any visible effect on the dielectric behavior, but it seems to provide suitable conditions for MoS_2_ nanosheets to make a conducting network inside the polymer blend. Similar work is also reported by Khan et al. [49] in which they have selected large aspect ratio nanofillers in polymer blends with low concentrations of PANI, resulting in high values of dielectric property.

### 3.3. EMI Shielding Efficiency of Hybrid Polymer Composites

The property of a material to either absorb or reflect the incoming EM wave from interacting with the electronic devices or to reduce its harmful effects is known as EMI shielding. The total shielding efficiency can be represented by the following expression:

*SE_T_* = *SE_A_* + *SE_R_* + *SE_I_* = 10log (I_IN_/I_out_) (5)

The above expression shows that the total shielding efficiency can be divided into three sections, i.e., absorption (*SE_A_*), refection (*SE_R_*), and multiple interaction (*SE_I_*) shielding efficiency, where *I_IN_* and *I_out_* are the incident and the transmitted EM waves thorough the shielding material, respectively. SE_I_ multiple interaction shielding efficiency is a positive or negative correction term, which is usually neglected when reflection shielding efficiency *SE_R_* > 10 dB. The magnetic or electric dipoles present in the specimen facilitate absorption of the EM wave, later emitted as heat. It increases the EM shielding efficiency. The reflection shielding efficiency is related to the presence of charges, which helps in reflecting the EM waves back in to the atmosphere. To calculate the total shielding *SE_T_*, first *SE_A_* and SE_R_ are calculated using the following mathematical equations [28,50]:

*SE_A_*= 8.8*αl* (6)

(7) $${\mathrm{SE}}_{R}=20\log\;\frac{{\left|1+n\right|}^{2}}{4\left|n\right|}\;$$

(8) $${\mathrm{SE}}_{I}=20\log\left|1-\;\frac{\exp{\left(-2\gamma l\right)}^{2}}{{\left(1+n\right)}^{2}}\right|$$

where *l* shows the thickness of the specimen, and *α*, *n*, and *γ* are the parameters, which are calculated by the following equations:

(9) $$\alpha \;=\;\frac{2\pi }{\lambda }\sqrt{\frac{\varepsilon \text{′}\sqrt{\left(1+tan^{2}\delta \right)}}{2}}$$

(10) $$n=\;\sqrt{\frac{\varepsilon \text{′}\sqrt{(1+tan^{2}\delta \;\pm \;1}}{2}}\;+\;i\sqrt{\frac{\varepsilon \text{′}\sqrt{(1+tan^{2}\delta \;\pm \;1}}{2}}$$

(11) $$\gamma =\;(\frac{2\pi }{\lambda })\sqrt{\frac{\varepsilon \text{′}\sqrt{(1+tan^{2}\delta \;\pm \;1}}{2}}\;+\;i(\frac{2\pi }{\lambda })\sqrt{\frac{\varepsilon \text{′}\sqrt{(1+tan^{2}\delta \;\pm \;1}}{2}}$$

In the above equations, *ε*′ and tan *δ* are the dielectric constant and dissipation factor of the shielding material, respectively, where λ is the wavelength of the incoming EM wave. In order to balance the positive and negative values of dielectric constant, the ± sign is added to the above equations.

**Figure 7:**
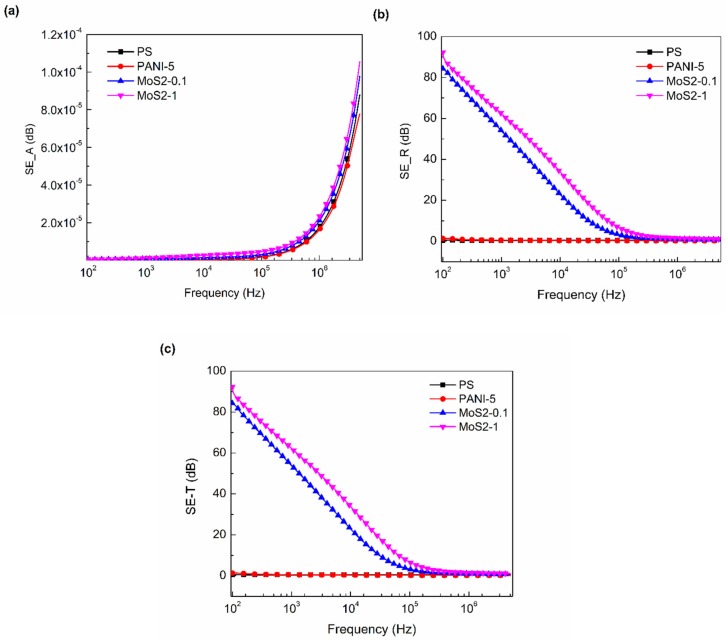
Electromagnetic interference (EMI) shielding behavior of PS, PANI, PS/PANI-5 wt % polymer blend, and PS/PANI/MoS_2_ hybrid polymer composites at various concentrations of MoS_2_ on the basis of (**a**) absorption shielding effectiveness (*SE_A_*), (**b**) reflection shielding efficiency (*SE_R_*), and (**c**) total shielding efficiency (*SE_T_*), as a function of frequency.

Figure 7a–c shows the theoretical EMI shielding response calculated from Equations (6)–(8) of pure PS polymer, PS/PANI-5 polymer blend, and PS/PANI-5/MoS_2_, with respect to the alternating frequency. At lower frequency range, absorption shielding efficiency (*SE_A_*) shows no increase, but as the frequency increases, the *SE_A_* also increases, as shown in Figure 7a. However, for reflection shielding efficiency (*SE_R_*), an increasing trend is observed at lower frequency range, and it reduces to lower values as the frequency is increased (Figure 7b). As for total shielding efficiency (*SE_T_*), Figure 7c shows pure PS, polymer blend, and hybrid polymer composites following the similar trend of reflection shielding efficiency (*SE_R_*), as there is no significant increase in *SE_A_*.

The addition of MoS_2_ at 0.1 wt % concentration in PS/PANI-5 polymer blend increases the shielding effectiveness to 84.2 dB, whereas a maximum value of 92.3 dB of SE_T_ is observed by adding 1 wt % concentration of MoS_2_ in PS/PANI-5 polymer blend at 100 Hz. The addition of MoS_2_ nanosheets to PS/PANI-5 polymer blend probably causes it to form a conductive network, which enables the material to maximally attenuate the EM waves and increase the shielding efficiency [51]. Furthermore, the highest values of *ε*′, *ε*″, σ_AC_, and tan *δ* are also noted at the same concentration of MoS_2_ inside the polymer blend, which further validates the increase in total shielding efficiency. Similar results were also reported by Gou et al. [52] in which a maximum reflection loss of −43 dB at 14.48 GHz was obtained by adding 5 wt % concentration of rGO@MoS_2_ composite in PVDF polymer matrix. The samples of rGO@MoS_2_/PVDF hybrid polymer composites were prepared by a solution casting technique. Moreover, Shah et al. [53] theoretically estimated (using dielectric behavior of samples) a total shielding efficiency of 208 dB for polyetherimide polyaniline-crafted few-layer graphene at 1.5 wt % few layer graphene (FLG) loading at 100 Hz. The samples were prepared by a solution casting technique. The thickness of the sample has an important role in enhancing the shielding efficiency. At a thickness of approximately 0.15 mm, promising results are shown by the PS/PANI-5/MoS_2_ hybrid polymer composite, which can be further increased to enhance the shielding efficiency. Our work is based on theoretical formulations that present highly approximated results, but still it provides an indication for further experimentations. The current work may pave the way for utilization of MoS_2_ and other 2D nanosheets for EMI shielding purposes in hybrid polymer composites.

## 4. Conclusions

Polystyrene, polyaniline, and MoS_2_-based hybrid polymer composites are prepared for dielectric characterization in the frequency range of 100 Hz to 5 MHz. Dielectric constant and loss are enhanced to 1.6 × 10^4^ and 1.6 × 10^5^, respectively, as compared to the PS-only values of 1.83 and 0.022 at 100 Hz. AC conductivity was also increased (100 Hz) from 3.42 × 10^−9^ S/cm for PS only to 8.98 × 10^−4^ PS/PANI (5 wt %)/MoS_2_ (1 wt %) composite. These dielectric characteristics are then translated in to theoretical EMI shielding effectiveness. With the inclusion of MoS_2_ nanosheets in the PS/PANI blends, the maximum estimated EMI shielding effectiveness reaches up to 92 dB at 100 Hz. While these values are highly approximated, it will be interesting to see the experimental results for these hybrid polymer composites in any frequency regime.

## Figures and Tables

**Table 1:** The values of *ε*′,*ε*″ and σ_AC_ for PS, PANI, PS/PANI-5wt% polymer blend, PS/PANI/MoS_2_ hybrid composites at 100 Hz.

Samples	100 (Hz)		AC Conductivity
	*ε′*	*ε″*	σ_AC_ (S/cm)
PS	1.83	0.022	3.422 × 10^−9^
PS/PANI-5 wt %	2.76	2.03	1.076 × 10^−8^
MoS_2_ (0.1 wt %)	8.5 × 10^3^	7.2 × 10^4^	4.243 × 10^−4^
MoS_2_ (1 wt %)	1.6 × 10^4^	1.6 × 10^5^	8.98 × 10^−4^
